# A New Professorship

**Published:** 1872-06

**Authors:** 


*A New Professorship.*—The Faculty of the Baltimore College of Dental Surgery have established a new professorship of "Clinical Dentistry," in addition to the chairs already existing, and appointed James H. Harris, D. D. S., late Demonstrator of Operative Dentistry, to the position, whose duty it will be to deliver clinical lectures at stated times during the session, and superintend the infirmary and laboratory practice. Dr. William Farmer, a Graduate of the Class of 1867, has been appointed Demonstrator of Operative Dentistry, in the place of Dr. Harris, promoted.

It is the object of the Faculty of this School, to present as complete a course of instruction in the practical departments, as will insure to all the students the greatest facilities for acquiring practical knowledge and manipulative dexterity.

